# Comparison of the acute physiological and perceptual responses between resistance-type and cycling high-intensity interval training

**DOI:** 10.3389/fphys.2022.986920

**Published:** 2022-09-09

**Authors:** Jun Mao, Tao Wang, Li Zhang, Qing Li, Shumin Bo

**Affiliations:** ^1^ College of Kinesiology and Health, Capital University of Physical Education and Sports, Beijing, China; ^2^ School of Physical Education, Liaocheng University, Liaocheng, China

**Keywords:** high-intensity interval training, resistance-type HIIT, cardiorespiratory response, heart rate variability, acute physiological response

## Abstract

**Purpose:** The purpose of the present study was to compare the acute physiological and perceptual responses between resistance-type high-intensity interval training (R-HIIT)and cycling high-intensity interval training (C-HIIT).

**Methods:** Twelve healthy and active men randomly performed C-HIIT and R-HIIT. The C-HIIT protocol was performed on a cycle ergometer and consisted of ten 60 s working intervals at 90% PPO separated by a 60 s active recovery at 25% PPO. The R-HIIT protocol consisted of ten 60 s working intervals (barbell back squat with a load of 20% bodyweight, maximum 30 reps) separated by 60 s passive recovery period in an unloaded standing position. Oxygen consumption ( $\dot{V}$ O_2_), heart rate (HR), energy expenditure (EE) and rating of perceived exertion (RPE) were measured during exercise. Blood lactate concentration (Blac), serum testosterone and cortisol, and heart rate variability (HRV) were measured before and after exercise.

**Results:** Peak (*p* < 0.05) and average $\dot{V}$ O_2_ (*p* < 0.001), aerobic (*p* < 0.001) and total EE (*p* < 0.05) were higher during C-HIIT compared to R-HIIT. Blac after exercise (*p* < 0.05) and anaerobic glycolytic EE (*p* < 0.05) during exercise were higher in R-HIIT compared to C-HIIT. No differences (*p* > 0.05) in peak and average HR, serum testosterone and cortisol, HRV, and RPE responses were observed between C-HIIT and R-HIIT.

**Conclusion:** The R-HIIT protocol can elicit similar cardiovascular, hormones, and perceptual responses as C-HIIT but with a higher contribution to the anaerobic glycolysis energy system. In contrast, C-HIIT is superior to R-HIIT for increasing oxygen consumption during exercise. Therefore, the two types of HIIT may lead to different metabolic and neuromuscular adaptations.

## 1 Introduction

Lack of physical activity or exercise is a major cause of most chronic diseases (Booth et al., 2012). As mentioned in the World Health Organization guidelines, adults should regularly spend at least 150–300 min on moderate-intensity or 75–150 min on vigorous-intensity, or engage in combination of moderate to vigorous physical activity throughout the week resulting in an equivalent level. This can reduce the incidences of chronic diseases (World Health Organization, 2018). High-intensity interval training (HIIT) has become a popular exercise modality worldwide (Thompson, 2022) and is characterized by repeated vigorous exercise at near-maximal workloads (≥ 85% HRmax or ≥ 80% $\dot{V}$ O_2_max) interspersed with active or passive recovery periods (MacInnis and Gibala, 2017). Multiple studies have shown that HIIT can produce similar (Wewege et al., 2017; Costa et al., 2018; Dias et al., 2018; Ryan et al., 2020) or even better (Milanović et al., 2015; Ramos et al., 2015) cardiovascular and metabolic benefits than traditional moderate-intensity continuous training (MICT). HIIT is typically performed on treadmills or cycle ergometers. However, in the past few years, resistance-type HIIT (R-HIIT) has drwan a lot of attention from both the recreationally active population and researchers (Falz et al., 2019; Bellissimo et al., 2022; Järvinen et al., 2022). R-HIIT is performed using resistance exercises, including free weights (barbells, dumbbells, or kettlebells), specialized equipment or body weight. A few studies have reported that R-HIIT can simultaneously improve cardiorespiratory fitness and muscle fitness (McRae et al., 2012; Buckley et al., 2015; Sheykhlouvand et al., 2022). Thus, R-HIIT can be considered a worthy exercise modality for people who aim to achieve higher health benefits in a fixed amount of time.

R-HIIT is generally performed in a circuit manner that alternately stimulates different muscle groups using multiple resistance exercises. However, different resistance exercises produce different levels of stimulation of the cardiorespiratory systems. This causes large fluctuations in cardiorespiratory responses throughout the exercise and is lower than in traditional HIIT (Riegler et al., 2017; Schleppenbach et al., 2017; Bellissimo et al., 2022; Järvinen et al., 2022). In a study by Järvinen et al. (Järvinen et al., 2022), they found that performing R-HIIT involving whole-body muscle groups, oxygen uptake was lower when the movement mainly engaged upper limbs and trunk muscle groups, while oxygen uptake was highest when lower limb muscle groups were highly engaged. Additionally, inconsistent cardiorespiratory responses were also elicited by different lower extremity movements in R-HIIT (Bellissimo et al., 2022). Therefore, we hypothesize that R-HIIT using a single lower extremity dynamic exercise can continue to increase cardiorespiratory response in a similar way to that seen with traditional HIIT. The squat is a classic resistance training exercise that involves neuromuscular and movement patterns similar to numerous sports and daily activities. As a result, it can enhance the athletic performance of athletes or improve the health and quality of life of the general population. (Schoenfeld, 2010). An initial study by Takai et al. (2013) showed that 8 weeks of bodyweight squats significantly reduced body fat and increased lean body mass, muscle thickness, muscular strength of the knee extensors, and jump performance in adolescent boys. Another study by Falz et al. (2019) compared the acute physiological responses of cycling MICT, HIIT, and resistance-type HIIT. They found that among the five bodyweight movements (squats, push-ups, isometric back extension, isometric leg raise, and inverted rows), only squats could elicit comparable stroke volumes as traditional HIIT. Based on the above studies, we hypothesized that squat-based R-HIIT might achieve the same cardiorespiratory response as traditional cycling HIIT.

Exercise can disrupt physiological homeostasis and cause significant hormonal changes. The appropriate endocrine response elicited by exercise can cause beneficial adaptations in the body, thereby helping in enhancing physical health as well as sports performance (Hackney and Lane, 2015). Testosterone and cortisol have been identified as the anabolic and catabolic hormonal responses to exercise-induced physiological stress, respectively (Adlercreutz et al., 1986). However, few studies have reported that different exercise modalities elicit different hormonal responses (Harris et al., 2018; Velasco-Orjuela et al., 2018). Therefore, the acute changes in testosterone and cortisol levels may vary between resistance-type HIIT and cycling HIIT because of differences in muscle activity and metabolism characteristics.

The autonomic nervous system (ANS) increases cardiac activity and controls hemodynamics by reducing parasympathetic nerve activity and simultaneously diverting sympathetic innervation during exercise. It also regulates the cardiovascular system to promote recovery after exercise (Borresen and Lambert, 2008). It is important to consider the time required for recovery of ANS after the workout, in addition to the response of the cardiovascular system during exercise. A better understanding of the recovery duration indicated by cardiac parasympathetic reactivation following exercise can contribute to improved programming of training sessions that aim to induce metabolic and cardiorespiratory adaptations (Stanley et al., 2013). Changes in cardiac parasympathetic activity can be assessed rapidly and non-invasively by measuring heart rate variability (HRV) after exercise (Malik, 1996). However, there are limited studies comparing the differences in HRV responses between resistance-type HIIT and cycling HIIT.

The acute response and long-term adaptation of physiological systems could be affected by the combinations of different training variables (work/recovery ratios, intensity and duration of the work/recovery periods) in HIIT (Buchheit and Laursen, 2013). The cycling HIIT pattern used in this study was 60:60 s with a sub-maximum load. This pattern is considered safe and effective in improving the health and fitness of varied populations of adults, including those who are active (Raleigh et al., 2016), sedentary (Hood et al., 2011), and overweight/obese (Boyd et al., 2013). The purpose of this study was to compare the acute oxygen consumption ( $\dot{V}$ O_2_), heart rate (HR), blood lactate concentration (Blac), serum testosterone, cortisol, heart rate variability, and rating of perceived exertion (RPE) between resistance-type high-intensity interval training (R-HIIT) and cycling high-intensity interval training (C-HIIT). We hypothesized that the R-HIIT protocol would elicit sustained elevated but lower cardiorespiratory responses ( $\dot{V}$ O_2_ and HR) and greater RPE, Blac, serum testosterone and cortisol, and HRV responses than C-HIIT.

## 2 Materials and methods

### 2.1 Participants

Eighteen recreationally active male participants were initially recruited from Capital University of Physical Education and Sports (Haidian District, Beijing, China). The inclusion criteria were: aged between 18 and 28 years, performing structured exercise training at least twice a week (≥ 150 min of moderate-intensity or 75 min of vigorous-intensity/week). None of the subjects had previously participated in systematic resistance training or high-intensity interval training. The exclusion criteria were: any disease, injury, or other condition that could compromise the ability to perform the exercise. Since the six subjects did not meet the requirements, 12 subjects (21.9 ± 2.6 years) were included in the study. The height, body mass, percentage of body fat, and body mass index were 178.5 ± 4. 5 cm, 72.4 ± 6.0 kg, 18.2 ± 6.1%, and 22.2 ± 1.3 kg/m^2^, respectively (Table 1). Prior to study initiation, the participants were fully informed of the study procedures, and all of them signed a written informed consent form. The minimal sample size of 12 was determined by *a priori* analyses using G*Power software (version 3.1.9.2) based on the following parameters: an alpha level of 0.05, a power (1-beta) equal to 0.8, and an effect size of 0.4.

**TABLE 1:** Anthropometric and physiological data for participants (*n* = 12).

Parameter	Mean ± SD
Age (years)	21.9 ± 2.6
Height (cm)	178.5 ± 4.5
Weight (kg)	72.4 ± 6.0
BF (%)	18.2 ± 6.1
BMI (kg/m^2^)	22.2 ± 1.3
$\dot{V}$ O_2_max (ml/kg/min)	45.9 ± 5.1
HRmax (bpm)	190.8 ± 15.9

Cm, centimeters; kg, kilograms; BF, body fat percentage; BMI, body mass index; $\dot{V}$ O_2_max, maximum oxygen consumption; ml, milliliters; min, minute; bpm, beats per minute. Data are displayed as means ± standard deviation.

### 2.2 Experimental design

**FIGURE 1:**
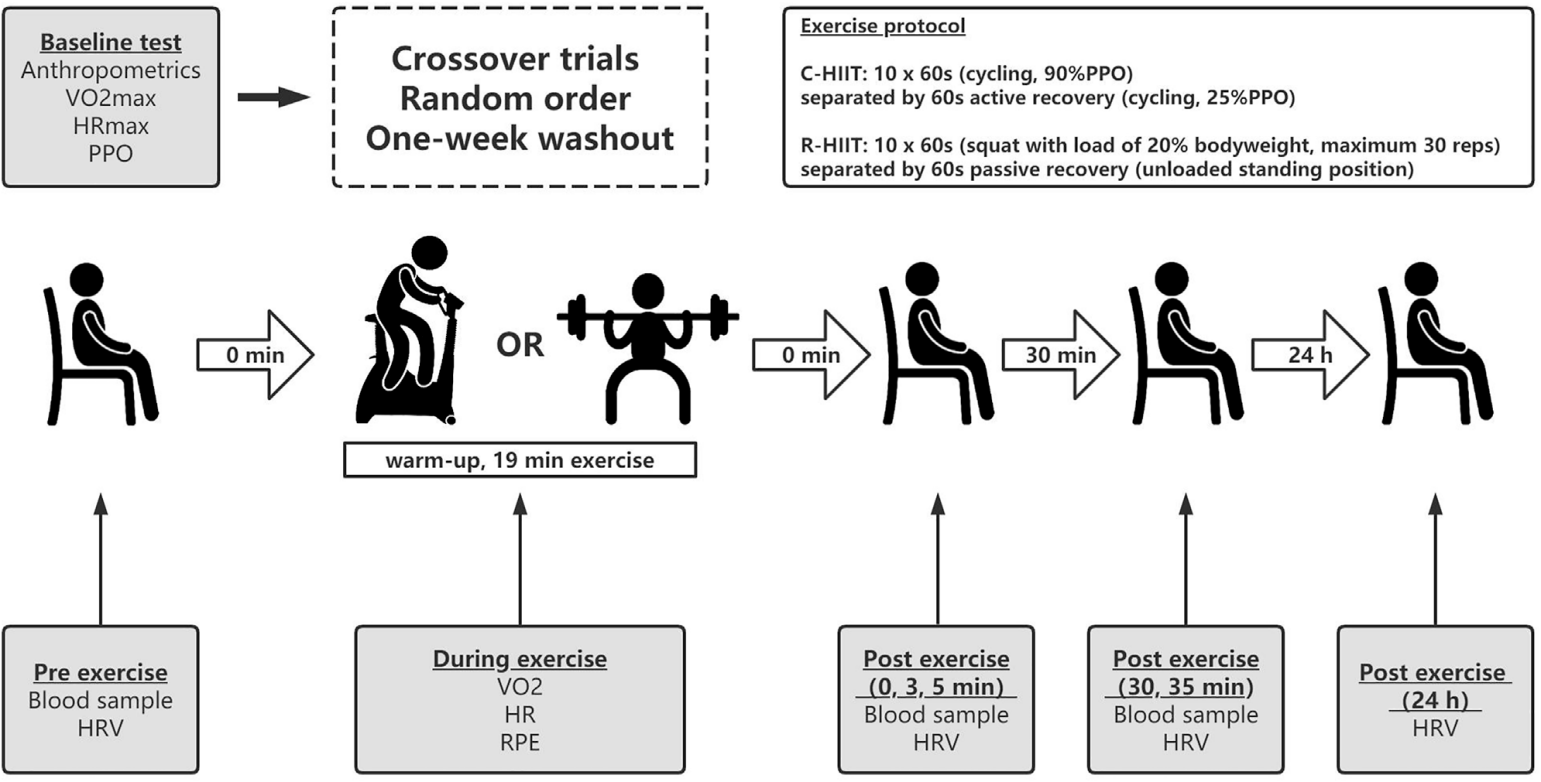
Experimental design of the study. C-HIIT, cycling high-intensity interval training; R- HIIT, resistance-type high-intensity interval training; PPO, peak power output; $\dot{V}$ O_2_, oxygen consumption; HR, heart rate; RPE, rating of perceived exertion; HRV, heart rate variability.

The current study employed a randomized crossover design. The experiments consisted of five visits to the laboratory. All visit sessions were separated by at least 72 h and at least 1 week washout period between two HIIT sessions. During the first visit, participants accepted anthropometric measurements and a maximal incremental cycling test to determine $\dot{V}$ O_2_max, HRmax, and the peak power output (PPO). They were then familiarized with the experimental procedure and resistance movements. In visits two to five, the participants performed two HIIT protocols in random order and were measured before, after and 24 following each exercise bout (Figure 1). Each exercise session consisted of a 5 min warm-up, 19 min of exercise intervention, and 30 min of sitting after exercise. All subjects completed the baseline tests and exercise sessions from 9:00 a.m. to 13:00 a.m. of the day. All subjects abstained from caffeine and alcohol intake for 24 h and did not perform any vigorous activity at least 48 h before each visit. Each subject consumed breakfast 2 h before testing began. The food intake before the visit was standardized; all subjects were instructed to record their food intake 24 h before the baseline test and then replicate their diet before and after 24 h each subsequent visit. All exercises and measurements were performed in a temperature-controlled (22–24°C) and humidity-controlled (40–50%) room. Ethical approval was granted by the Capital University of Physical Education and Sports Ethical Committee. The trial was registered in the Chinese Clinical Trial Registry (www.chictr.org.cn, registration number: ChiCTR2200056897).

### 2.3 Baseline testing

Participants’ height (cm) and bodyweight were measured using an ultrasonic tester (DHM-200, China) and BMI was calculated by dividing the subjects’ weight (kilograms) by the square of their height (meters). Body composition was assessed by dual x-ray absorptiometry (Lunar, United States). All anthropometric data are shown in Table 1.

$\dot{V}$ O_2_max was determined during a maximal incremental cycling test performed on a cycling ergometer (ergoline 100 K, Germany). The subjects maintained a pedalling cadence between 60–65 revolutions per minute (rpm) throughout the entire test. After a 5-min warm-up at 50 W, power was increased by 15 W from 70 W every minute until volitional exhaustion (operationally defined as a cadence of <60 revolutions/min for >5 s, despite strong verbal encouragement). Expired gases were analyzed using a gas analyzer (AEI moxus, United States), and HR was recorded simultaneously using a coded transmitter belt (Polar H10, Finland). The rate of perceived exertion (RPE, 6–20 Borg’s scale) was determined after each stage and at the end of the test. Participants were considered to have reached their $\dot{V}$ O_2_max when at least three of the four following criteria were met: 1) a plateau of $\dot{V}$ O_2_ (i.e., change ≤2.1 ml/kg/min), 2) a final respiratory exchange ratio (RER) ≥ 1.1, 3) maximum heart rate within 10 bpm of the age-predicted maximum [210-(0.65 × age)], and 4) RPE ≥18. $\dot{V}$ O_2_max was defined as the mean of the highest values obtained during three consecutive 10-s periods (30 s in total) and are presented in Table 1.

Peak power output (PPO) was identified at the work rate coincident with volitional fatigue. When the exercise intensity could not be maintained for the full 60 s, PPO was calculated using the equation (Kuipers et al., 1985):

$$PPO=W_{final}+\left(t/T\right)•W_{inc}$$

PPO is the peak power output (W), W_final_ is the last power output (W) completed for 60 s, t (s) is the amount of time reached in the final uncompleted stage, T (s) is the duration of each stage (60 s), and Winc (W) is the workload increment (15 W).

### 2.4 Exercise protocol

The participants performed two exercise sessions in random order and on two separate occasions (Figure 1). Participants were given verbal encouragement during both HIIT protocols. In our pilot experiment, we matched the intensity of the two HIIT conditions by assessing the perceived effort levels directly after exercise. This method has been used by other researchers (Rønnestad et al., 2020; Järvinen et al., 2022).

Before starting C-HIIT, participants performed approximately 10 min of warm-up, including dynamic stretches and 3 min cycling at 50 W. The warm-up was followed by a 1-min transition period before the start of the session. The C-HIIT protocol was performed on a cycle ergometer (ergoline 100 K, Germany) and consisted of ten 60 s working intervals at 90% PPO interspersed with a 60 s active recovery at 25% PPO. During the exercise, subjects were instructed to maintain pedal cadence between 60–65 revolutions per minute.

Before starting R-HIIT, participants performed approximately 10 min of warm-up, including dynamic stretches and squats without load. The R-HIIT protocol consisted of ten 60 s working intervals (barbell back squat with a load of 20% bodyweight) separated by 60 s recovery periods (put down the loaded barbell and rest in a standing position). The participant placed the barbell on the trapezius with the assistance of the researcher and held the barbell in both hands. During all repetitions, participants were asked to squat down to approximately 90° knee flexion, the feet were required to maintain contact with the floor (e.g., no jumping or lifting of the heels), and full hip and knee extension was required at the conclusion of each repetition. They were instructed to perform the eccentric phase of each squat for 1 s while performing the concentric phase of each squat as fast as possible to a standing position. The maximum number of squats completed per working interval is 30 reps. A metronome is used to control the tempo of the movements (60 beats/min). If the participant is unable to complete the movement at the prescribed tempo, a brief rest in a standing position with load is allowed. The researcher removed the barbell immediately after each working interval and repositioned the barbell in the participant’s trapezius 5 s before the next exercise bout.

### 2.5 Measurement

#### 2.5.1 Oxygen consumption, heart rate, energy expenditure, and perceptual responses

During exercise, all $\dot{V}$ O_2_ data were collected using a gas analyzer (AEI moxus, United States), and heart rate data were collected using a chest belt (Polar H10, Finland). During the entire exercise session, $\dot{V}$ O_2_ and HR data were obtained every 10 s. Peak $\dot{V}$ O_2_ and HR were defined as the mean of the highest values obtained during three consecutive data points. Mean $\dot{V}$ O_2_ and HR were defined as the average value acquired from the entire session (114 data points) excluding the warm-up. The last data points of every minute were taken to analyze different time points during the exercise. $\dot{V}$ O_2_ relative to $\dot{V}$ O_2_max (% $\dot{V}$ O_2_max) and HR relative to HRmax (%HRmax) during each HIIT protocol were calculated. The caloric equivalent was set at 5.05 kcal/L during the whole exercise period. Anaerobic glycolytic energy expenditure (EE) was estimated using the O_2_ equivalent of blood lactate accumulation. Δ[La] was calculated by subtracting resting values from peak [La] reached 3 min after exercise. Measures of Δ[La-] were converted to equivalent values as 3 ml O_2_ kg^−1^ bodyweight per mmol of Δ[La-] (di Prampero and Ferretti, 1999). Rating of perceived exertion (RPE) was recorded immediately after each working interval using the Borg CR10 Scale; the subjects were familiarized with the RPE scale at the first visit and expressed their scores using hand gestures during exercise.

#### 2.5.2 Blood lactate concentration, testosterone, and cortisol levels

Blood lactate concentration from fingertip blood sample was measured with a portable device (Lactate Scout, H/P COSMOS, Germany) before warm-up, 3 min after exercise, and 30 min after exercise. Venous blood samples were collected before the warm-up, immediately after exercise, and 30 min after exercise for the determinutesation of testosterone and cortisol levels. The whole blood samples were allowed to clot at room temperature for 30 min, then centrifuged at 3,000 rpm for 10 min at 4°C. The separated serum samples were frozen and kept at −80°C. The serum testosterone and cortisol levels were measured with the radioimmunoassay method (XH6080, Xi’an Nuclear Instrument Factory, China).

#### 2.5.3 Heart rate variability

All variables related to HRV were assessed in 4 moments: 1) before warm-up, 2) 5 min after exercise, 3) 35 min after exercise, and 4) 24 h after exercise. Autonomic nervous system activity in HRV was measured by an electrocardiographic workstation (Shenzhen Boying Medical Instrument Technology Co., China). Electrocardiogram electrodes were placed on their wrists with the subjects in the supine position. The values of every R-R interval were obtained by the system completely independent of human operation, and 5 min were selected for analysis. For the HRV data processing, the Kubios software (v.3.5.0, HRV analysis, Finland) (Tarvainen et al., 2014) was used. The time-domain variable of HRV used in the present analysis was the root mean square difference of successive normal R-R interval (RMSSD). The frequency domain variable was high-frequency power of 0.15–0.40 Hz (HF). Because the raw values of RMSSD and HF were skewed toward higher values and violated assumptions of normality, natural log(ln) transformation was applied (lnRMSSD and lnHF).

#### 2.6 Statistical analyses

Data are presented as mean ± standard deviation (SD). Statistical analyses were performed with SPSS Statistics (version 26, IBM, United States). Shapiro–Wilk tests were used to check normality assumptions. The alpha level was set at a value of *P* of <0.05 for indication of statistical significance for all analyses. Two-tailed paired sample t-tests were used to identify statistical differences in measurements of peak or average of $\dot{V}$ O_2_, HR, EE, and RPE between conditions. Two-factor (condition × time) repeated measures ANOVA was used to examine the effects of exercise and time on $\dot{V}$ O_2_, HR, RPE, Blac, testosterone, cortisol, lnRMSSD, and lnHF. Bonferroni’s correction factor was employed to test multiple comparisons. Partial eta squared (η^2^) was calculated to determine the magnitude of main and interaction effects and was categorized as small (0.01), medium (0.06), and large (0.14), respectively. Cohen’s d was used as a measure of effect size, with a small, medium, and large effect equal to 0.2, 0.5, and 0.8, respectively (Cohen, 1992).

## 3 Results

### 3.1 Oxygen consumption ( $\dot{V}$ O2)

**FIGURE 2:**
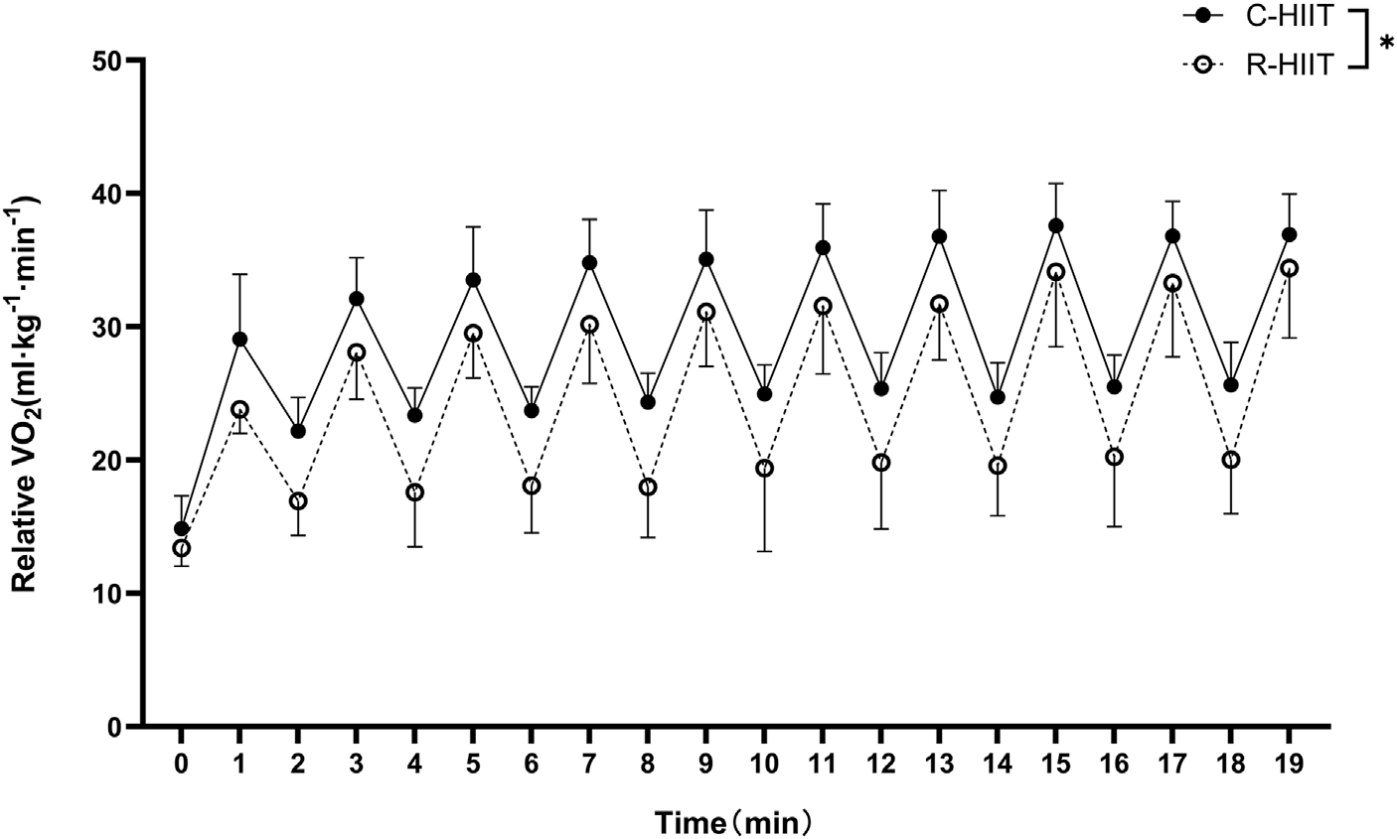
$\dot{V}$ O_2_ response during C-HIIT and R-HIIT exercise sessions, *p < 0.05 C-HIIT was significantly higher than R-HIIT during exercise.

$\dot{V}$ O_2_peak, $\dot{V}$ O_2_peak (%), $\dot{V}$ O_2_mean, and $\dot{V}$ O_2_mean (%) measured during C-HIIT were significantly higher than during R-HIIT (*p* = 0.022, d = 0.59; *p* = 0.021, d = 0.89; *p* < 0.001, d = 1.61; *p* < 0.001, d = 1.97; Table 2). The changes in $\dot{V}$ O_2_ during the two exercise sessions are displayed in Figure 2. For all time points during exercise (0–19 min). No significant time × condition interaction effect was observed on $\dot{V}$ O_2_ (F = 1.466, *p* = 0.206, η^2^ = 0.062). A significant main effect was observed for exercise condition on $\dot{V}$ O_2_ (F = 17.707, *p* = 0.001, η^2^ = 0.417). A significant main effect was observed for time in which $\dot{V}$ O_2_ significantly increased across exercise (F = 179.663, *p* < 0.001, η^2^ = 0.981).

**TABLE 2:** $\dot{V}$ O2, HR, RPE, and EE values during exercise.

	C-HIIT	R-HIIT	*p*-value	Cohen’s d
$\dot{V}$ O_2_peak (ml·kg^−1^ min^−1^)	38.9 ± 4.0^a^	36.4 ± 4.4	0.022	0.59
$\dot{V}$ O_2_peak (% $\dot{V}$ O_2_max)	85.1 ± 4.9^a^	79.5 ± 7.5	0.021	0.89
$\dot{V}$ O_2_mean (ml·kg^−1^ min^−1^)	29.6 ± 2.3^a^	25.1 ± 3.2	<0.001	1.61
$\dot{V}$ O_2_mean (% $\dot{V}$ O_2_max)	64.8 ± 3.3^a^	55.0 ± 6.2	<0.001	1.97
HRpeak (beats·min^−1^)	178.0 ± 14.1	182.4 ± 18.4	0.218	0.27
HRpeak (%HRmax)	91.8 ± 5.0	93.9 ± 4.9	0.216	0.42
HRmean (beats·min^−1^)	154.2 ± 15.4	149.9 ± 17.1	0.070	0.26
HRmean (%HRmax)	79.4 ± 4.5	77.1 ± 5.1	0.069	0.48
RPEpeak (1–10)	7.25 ± 1.16	8.17 ± 0.90	0.059	0.89
RPEmean (1–10)	5.67 ± 1.19	5.63 ± 0.95	0.935	0.04
Energy expenditure (kcal)				
Aerobic	205.8 ± 24.0^a^	174.4 ± 22.8	<0.001	1.34
Anaerobic glycolytic	9.0 ± 1.8^a^	11.7 ± 2.2	0.002	1.34
Total	214.7 ± 23.5^a^	186.1 ± 23.8	0.001	1.21

a *p* < 0.05 significantly different between C-HIIT, and R-HIIT.

### 3.2 Heart rate (HR)

**FIGURE 3:**
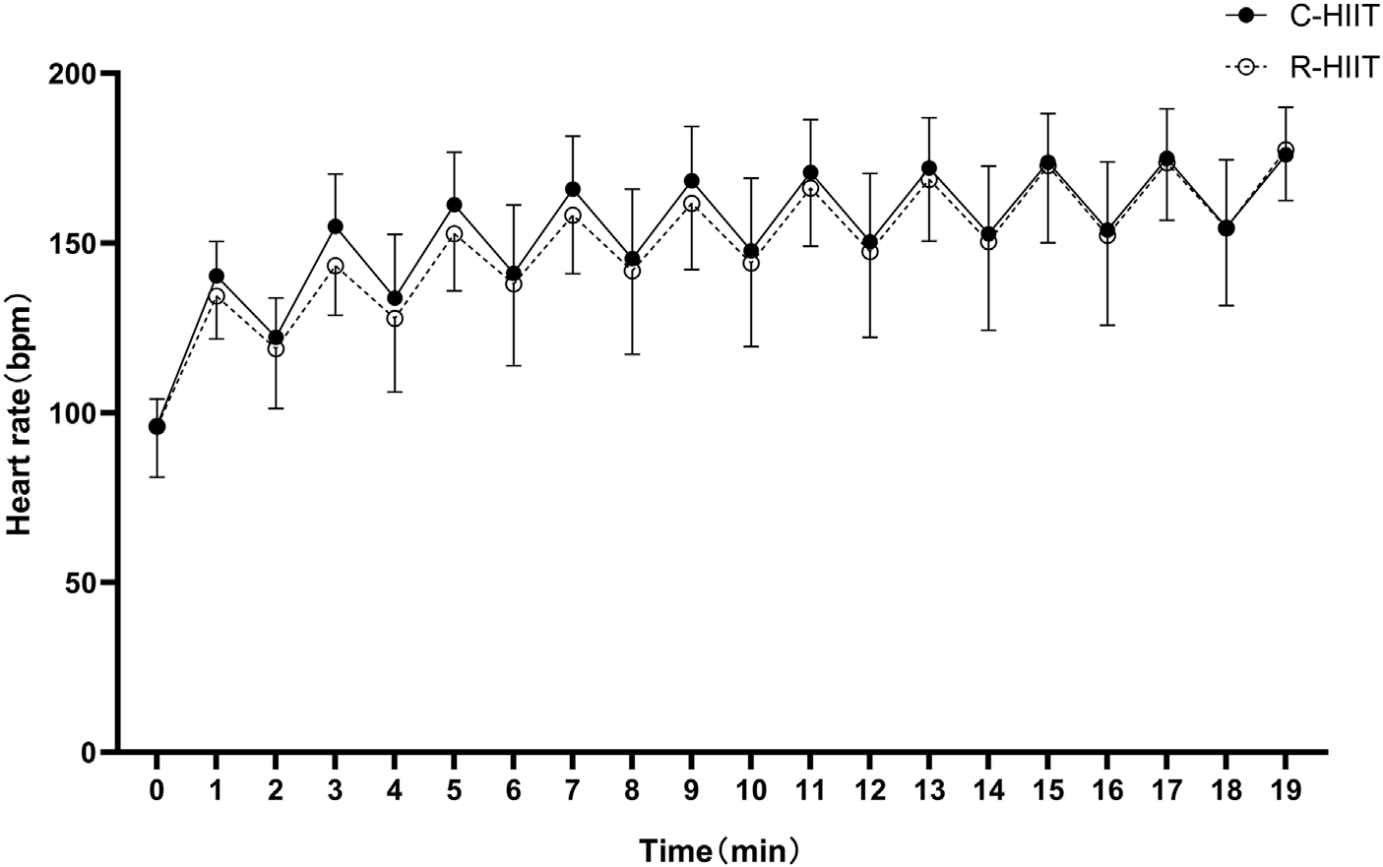
Heart rate response during C-HIIT and R-HIIT exercise sessions.

There was no significant difference in HRpeak, HRpeak (%), HRmean and HRmean (%) between C-HIIT and R-HIIT (*p* = 0.218, d = 0.27; *p* = 0.216, d = 0.42; *p* = 0.070, d = 0.26; *p* = 0.069, d = 0.48; Table 2). The changes in HR during the two exercise sessions are displayed in Figure 3. For all time points during exercise (0–19 min). No significant time × condition interaction effect was observed on HR (F = 0.906, *p* = 0.436, and η2 = 0.040). No significant main effect was observed for exercise condition on HR (F = 0.308, *p* = 0.584, and η^2^ = 0.014). A significant main effect was observed for time in which HR significantly increased across exercise (F = 141.184, *p* < 0.001, and η^2^ = 0.865).

### 3.3 Energy expenditure (EE)

Aerobic and total EE measured during C-HIIT was significantly higher than during R-HIIT (*p* < 0.001, d = 1.34; *p* = 0.001, d = 1.21; Table 2). Anaerobic glycolytic EE measured during R-HIIT was significantly higher than during C-HIIT (*p* = 0.002, d = 1.34; Table 2).

### 3.4 Rating of perceived exertion (RPE)

**FIGURE 4:**
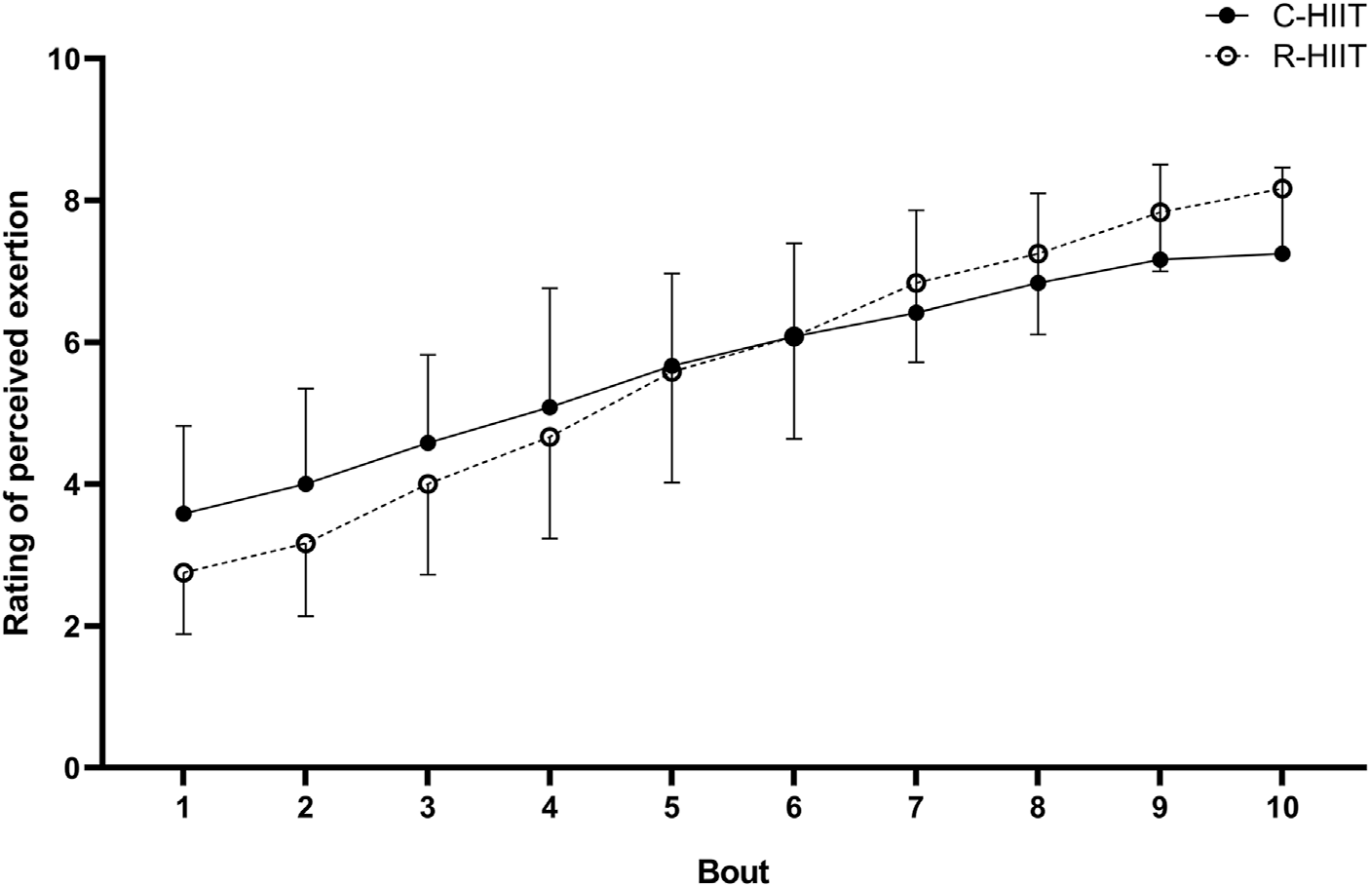
RPE response during C-HIIT and R-HIIT exercise sessions.

There was no significant difference in RPEpeak and RPEmean between C-HIIT and R-HIIT (*p* = 0.059, d = 0.89, *p* = 0.935, and d = 0.04; Table 2). The changes in RPE during the two exercise sessions are displayed in Figure 4. For all intervals during exercise (1–10 intervals). A significant time × condition interaction effect was observed on RPE (F = 6.233, *p* = 0.001, and η^2^ = 0.221). No significant main effect was observed for exercise condition on RPE (F = 0.005, *p* = 0.943, and η^2^ < 0.001). A significant main effect was observed for time in which RPE significantly increased across exercise (F = 166.795, *p* < 0.001, and η^2^ = 0.883).

### 3.5 Blood lactate, testosterone, and cortisol

**FIGURE 5:**
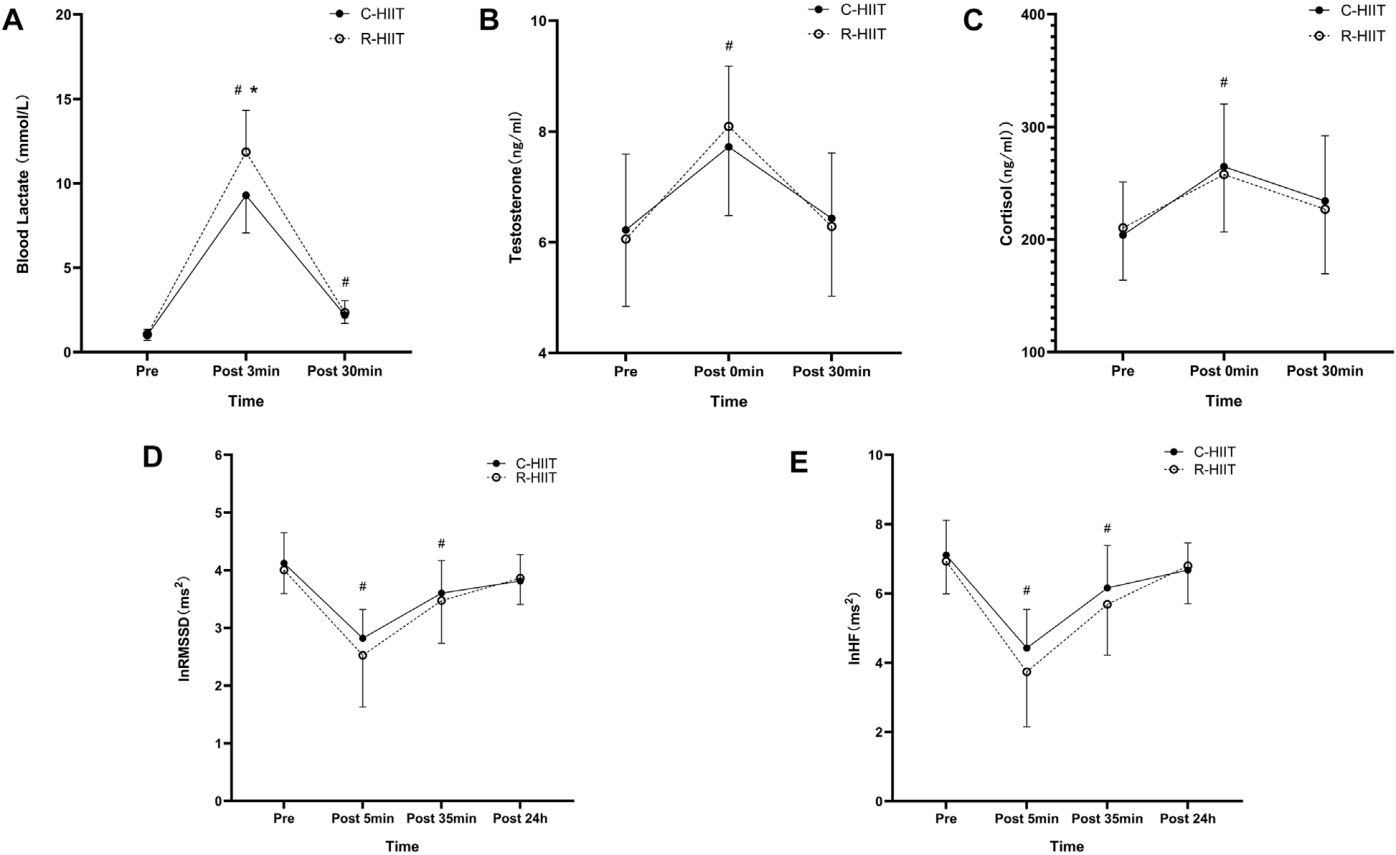
Blood lactate **(A)** at baseline (Pre), 3 min after exercise (Post 3 min), and 30 min after exercise (Post 30 min). Testosterone **(B)** and Cortisol **(C)** at baseline (Pre), immediately after exercise (Post 0 min), and 30 min after exercise (Post 30 min). lnRMSSD **(D)** and lnHF **(E)** at baseline (Pre), 5 min after exercise (Post 5 min), 35 min after exercise (Post 35 min), and 24 h after exercise (Post 24 h). *p < 0.05 R-HIIT was significantly higher than C-HIIT during exercise, # < 0.05 significantly different from Pre.

For blood lactate (Table 3; Figure 5A). A significant time × condition interaction effect was observed (F = 7.339, *p* = 0.010, and η^2^ = 0.250). A significant main effect was observed for exercise condition (F = 5.653, *p* = 0.027, and η^2^ = 0.204). A significant main effect was observed for time (F = 392.073, *p* < 0.001, and η^2^ = 0.947). For both exercise conditions, lactate increased above baseline after exercise (*p* < 0.001) and remained elevated through 30 min (*p* < 0.001). Blood lactate was significantly higher at 3 min post-exercise for R-HIIT than C-HIIT (*p* = 0.014, d = 1.15). No differences were observed for blood lactate at baseline (*p* = 0.685, d = 0.33) or 30 min after exercise (*p* = 0.552, d = 0.25) between C-HIIT and R-HIIT.

**TABLE 3:** Blood lactate, testosterone, cortisol, and HRV values before and after exercise.

	C-HIIT	R-HIIT	*P*		
			Interaction effect	Condition	Time
Blood lactate (mmol/L)					
Pre	1.0 ± 0.3	1.1 ± 0.3	0.010	0.027	<0.001
Post 3 min	9.3 ± 2.1#^a^	11.9 ± 2.4#
Post 30 min	2.19 ± 0.5#	2.34 ± 0.7#
Testosterone (ng/ml)					
Pre	6.2 ± 1.3	6.1 ± 1.2	0.735	0.970	<0.001
Post 0 min	7.7 ± 1.4#	8.1 ± 1.5#
Post 30 min	6.4 ± 1.1	6.3 ± 1.2
Cortisol (ng/ml)					
Pre	204.0 ± 45.1	210.3 ± 44.4	0.658	0.887	<0.001
Post 0 min	264.7 ± 53.2#	257.8 ± 48.9#
Post 30 min	234.3 ± 55.6	226.9 ± 54.9
lnRMSSD (ms^2^)					
Pre	4.1 ± 0.5	4.0 ± 0.4	0.578	0.505	<0.001
Post 5 min	2.8 ± 0.5#	2.5 ± 0.9#
Post 35 min	3.6 ± 0.5#	3.5 ± 0.7#
Post 24 h	3.8 ± 0.4	3.9 ± 0.4
lnHF (ms^2^)					
Pre	7.1 ± 1.0	6.9 ± 0.9	0.472	0.531	<0.001
Post 5 min	4.4 ± 1.1#	3.9 ± 1.4#
Post 35 min	6.2 ± 1.2	5.8 ± 1.4
Post 24 h	6.7 ± 0.8	6.8 ± 1.1

a *p* < 0.05 significantly different between C-HIIT, and R-HIIT, #*p* < 0.05 significantly different from pre.

For testosterone (Table 3; Figure 5B). No significant time × condition interaction effect was observed (F = 0.309, *p* = 0.735, and η^2^ = 0.014). No significant main effect was observed for exercise condition (F = 0.001, *p* = 0.970, and η^2^ < 0.001). A significant main effect was observed for time (F = 55.193, *p* < 0.001, and η^2^ = 0.418). For both exercise conditions, testosterone increased above baseline immediately after exercise (*p* < 0.05) and returned to baseline 30 min after exercise (*p* > 0.05). No differences were observed for testosterone at baseline (*p* = 0.756, d = 0.08), immediately after exercise (*p* = 0.564, d = 0.28) and 30 min after exercise (*p* = 0.776, d = 0.09) between C-HIIT and R-HIIT.

For cortisol (Table 3; Figure 5C). No significant time × condition interaction effect was observed (F = 0.352, *p* = 0.658, η^2^ = 0.016). No significant main effect was observed for exercise condition (F = 0.020, *p* = 0.887, η^2^ = 0.001). A significant main effect was observed for time (F = 17.172, *p* < 0.001, η^2^ = 0.715). For both conditions, cortisol increased above baseline immediately after exercise (*p* < 0.001) and returned to baseline 30 min after exercise (*p* > 0.05). No differences were observed for cortisol at baseline (*p* = 0.745, d = 0.12), immediately after exercise (*p* = 0.753, d = 0.14) and 30 min after exercise (*p* = 0.757, d = 0.13) between C-HIIT and R-HIIT.

### 3.6 Heart rate variability

For lnRMSSD (Table 3; Figure 5D). No significant time × condition interaction effect was observed (F = 0.589, *p* = 0.578, and η^2^ = 0.026). No significant main effect was observed for exercise condition (F = 0.460, *p* = 0.505, and η^2^ = 0.020). A significant main effect was observed for time (F = 44.152, *p* < 0.001, and η^2^ = 0.667). For both conditions, lnRMSSD dropped significantly from baseline at 5 and 35 min after exercise (*p* < 0.001) and returned to baseline 24 h after exercise (*p* > 0.05). No differences were observed for lnRMSSD at baseline (*p* = 0.564, d = 0.22), 5 min after exercise (*p* = 0.332, d = 0.41), 35 min after exercise (*p* = 0.633, d = 0.16) and 24 h after exercise (*p* = 0.791, d = 0.25) between C-HIIT and R-HIIT.

For lnHF (Table 3; Figure 5E). No significant time × condition interaction effect was observed (F = 0.789, *p* = 0.472, and η^2^ = 0.035). No significant main effect was observed for exercise condition (F = 0.046, *p* = 0.531, and η^2^ = 0.018). A significant main effect was observed for time (F = 65.510, *p* < 0.001, and η^2^ = 0.749). For both conditions, lnHF dropped significantly from baseline at 5 and 35 min after exercise (*p* < 0.001) and returned to baseline 24 h after exercise (*p* > 0.05). No differences were observed for lnHF at baseline (*p* = 0.661, d = 0.21), 5 min after exercise (*p* = 0.351, d = 0.40), 35 min after exercise (*p* = 0.477, d = 0.31) and 24 h after exercise (*p* = 0.752, d = 0.10) between C-HIIT and R-HIIT.

## 4 Discussion

In this study, we compared the acute physiological and perceptual responses between resistance-type high-intensity interval training (R-HIIT) and cycling high-intensity interval training (C-HIIT). The major findings were that C-HIIT induced greater oxygen consumption and energy expenditure while R-HIIT elicited higher blood lactate levels. In contrast to our hypothesis, no significant differences in heart rate, testosterone, cortisol, heart rate variability, and perceived exertion ratings in the two HIIT conditions.

Our findings confirmed that squat-based R-HIIT could consistently elevate the cardiorespiratory response as traditional cycling HIIT (Figures 2, 3). On the other hand, the results also showed that the peak and mean oxygen consumption of R-HIIT remained lower than that of C-HIIT (Table 2). This result is likely attributed to the greater recruitment of lower threshold motor units during C-HIIT that relies more on aerobic metabolism for adenosine triphosphate supply (Wood et al., 2016). In contrast, R-HIIT recruits more high-threshold motor units because of the need to overcome external load as the requirement of “as fast as possible in concentric contraction” that relies more on anaerobic metabolic systems. This speculation could be supported by higher blood lactate levels observed after R-HIIT exercise (Table 3). Moreover, the type of muscle contraction could also affect oxygen demand. For instance, the primary contraction type of the lower limb muscle groups in cycling is concentric contraction (Bijker et al., 2002), whereas the squatting movement involves eccentric contraction that requires less oxygen (Douglas et al., 2017). Another critical factor is the mode of recovery during the recovery period. R-HIIT uses passive recovery, whereas C-HIIT uses low-intensity active recovery (25% PPO) which is the traditional HIIT model commonly used in research and exercise practice. Active recovery can lead to greater deoxygenation of the exercising muscle groups than passive recovery, which causes higher oxygen demand (Fennell and Hopker, 2021). Therefore, the aerobic and total energy expenditure during exercise was significantly greater in C-HIIT than in R-HIIT. These results are also consistent with several previous studies comparing traditional HIIT with resistance-type HIIT (Schleppenbach et al., 2017; Järvinen et al., 2022).

Although the oxygen consumption of R-HIIT is lower than that of C-HIIT during exercise, the heart rate response is similar to that of C-HIIT (Figure 3). Similarly, other studies have also observed the differences between oxygen uptake and heart rate response. For instance, Bellissimo et al. (2022) compared bodyweight high-intensity interval exercise (HIIE) consisting of various plyometric-based calisthenics to treadmill running HIIE and found that there was less difference in the HR response (%HRpeak ∼5%) than the $\dot{V}$ O_2_ response (% $\dot{V}$ O_2_peak ∼11%). One of the reasons for this could be that R-HIIT recruited more fast muscle fibers and relied more on anaerobic metabolism for quick contractions. The greater stimulation of neurological receptors results in elevated heart rate levels (Gotshalk et al., 2004). In addition, the continuous postural change in the squatting movement will also induce a rapid increase in heart rate (Borst et al., 1982). Therefore, in order to achieve the same oxygen consumption as cycling HIIT, a higher heart rate is required when performing resistance-type HIIT. Despite the fact that the oxygen consumption response of R-HIIT was lower than that of C-HIIT, the oxygen consumption and heart rate levels of the R-HIIT condition in this study could still be classified as vigorous aerobic exercise (i.e., 64–90% of maximal oxygen uptake and 77–95% of maximum heart rate) in accordance with the ACSM guidelines (Garber et al., 2011).

In the R-HIIT condition, higher post-exercise blood lactate indicates a greater proportion of anaerobic glycolytic energy system supply. The primary reason is that the muscles must overcome a higher force with each contraction during the squat. Thus, the recruitment of more glycolytic muscle fibers and a more activated glycolytic system leads to a greater accumulation of blood lactate. Moreover, the strong force of muscular contraction might lead to an obstructed blood flow by compressing the blood vessels. Therefore, a reduction in blood flow to the exercising muscles most likely decreased the amount of oxygen being delivered to the mitochondria and reduced the elimination of metabolic end-products (Gotshalk et al., 2004). Additionally, the low-intensity active recovery used in the C-HIIT condition may have facilitated the clearance of blood lactate (Mandroukas et al., 2011). There is some evidence that exercise-induced metabolic stress is associated with acute hormonal responses (Gordon et al., 1994; Goto et al., 2005; Wahl et al., 2010). A previous study has shown that the decreased blood pH during exercise acts as a stimulus for exercise-induced cortisol secretion (Wahl et al., 2010). However, the two HIIT conditions in the present study elicited a similar change in cortisol levels. Our results align with a study done by Harris et al. (2018), where they found that full body low-load, high repetition resistance endurance training elicited higher blood lactate and growth hormone than a matched time and caloric cost cycling protocol but no significant difference in cortisol levels. Therefore, we presume that blood lactate concentrations in R-HIIT may not be sufficient to cause a greater change in cortisol levels. Additionally, no difference in testosterone levels was observed between the two HIIT protocols. The acute increase in testosterone levels can promote protein synthesis, leading to skeletal muscle hypertrophy and increased muscle strength (Vingren et al., 2010; Hooper et al., 2017). It is worth noting that HIIT can modulate the expression of genes and proteins involved in muscle mass regulation, increase muscle protein synthesis and activates muscle satellite cells (Callahan et al., 2021). Since squatting can recruit more and faster muscle fibers than cycling, it can be speculated that in the long term, R-HIIT can offer a greater advantage in building muscle and improving muscle strength.

Cardiac parasympathetic activity is suppressed and is fully recovered after at least 24 h of a single aerobic-based training session (Stanley et al., 2013). The present study showed that both HIIT conditions suppressed parasympathetic activity at 5 and 35 min after exercise and returned to baseline after 24 h with no significant difference. These observations are consistence with a study by Schaun and Del Vecchio (2018), where they compared bodyweight HIIT and cycling HIIT in the Tabata model. Since exercise intensity is an essential factor influencing heart rate variability (Parekh and Lee, 2005), the similar heart rate responses of R-HIIT and C-HIIT during exercise might explain the consistency of HRV change and recovery rate after exercise. From the perspective of the autonomic nervous system, these findings indicate that the participants fully recovered in 24 h after exercise and were able to conduct a subsequent exercise session. However, it is worth noting that the recovery of cardiac parasympathetic activity is not synchronized with other physiological systems. For example, the recovery of circulating muscle creatine kinase levels and protracted muscle pain lags behind the parasympathetic activity after weightlifting training (Chen et al., 2011). Most subjects in our study reported that R-HIIT elicited a greater degree of muscle soreness than C-HIIT 24 h after exercise. This could be attributed to the more severe muscle damage caused by the eccentric muscle contraction involved in the squatting movement (Allen, 2001). The delayed-onset muscle soreness may prevent the practitioner from exercising the next time or may limit the timing and frequency of subsequent sessions. Therefore, it is important for exercise instructors to schedule the exercise session according to the recovery levels of different physiology systems.

There was a clear linear increase in RPE after each working interval throughout both the C-HIIT and the R-HIIT conditions with no significant difference (Figure 4). Previous studies have reported that lower RPE is induced by bodyweight HIIT than by traditional cycling HIIT. Therefore, it can be speculated that alternately activating whole-body muscle groups can reduce local muscle stress compared to constant cycling (Gist et al., 2014; Riegler et al., 2017). In contrast, the squat exercise used in this study was similar to cycling, activating mainly the lower extremity muscle groups and resulting in similar RPE. Despite a gradual increase in RPE, all subjects maintained a defined rpm during the C-HIIT session. We propose that subjects might be able to complete more bouts at the defined rpm and intensity. However, in R-HIIT, not all subjects could complete the prescribed number of reps in all working intervals. Two subjects in the last three bouts and one subject in the last four bouts could not achieve the prescribed 30 reps. Unlike the other studies, the R-HIIT protocol did not use “all-out” as a requirement in the working interval but a fixed number of 30 reps. Thus, we believe it is similar to the “keeping rpm” in cycling and can delay the onset of fatigue. In the case of “as many reps as possible” requirements, the practitioner may accumulate fatigue quickly and perhaps gradually reduce the number of reps during the exercise (Bellissimo et al., 2022). Although there were no statistically significant difference in RPE between C-HIIT and R-HIIT, the higher blood lactate levels in R-HIIT after the last bout indicated that R-HIIT might have accumulated more fatigue. In addition, the greater change magnitude of RPE in R-HIIT might also suggest that fatigue was accumulated more rapidly than in C-HIIT (Figure 4). Excessive fatigue during resistance exercise can undermine correct movement techniques and increase the risk of injury (Trafimow et al., 1993). In order to avoid extreme fatigue during resistance-type HIIT, the exercise intensity could be reduced by changing the load, or/and changing the number of reps completed per working interval, or/and inserting extra rest periods. However, the change of exercise intensity perhaps leads the cardiovascular response would no longer reach the target range.

There are several limitations of the present study that should be acknowledged. First, the definition of HIIT has been used inconsistently. Previous studies have defined HIIT depend on the physiological response induced rather than the type of physical challenge or activity. In this study, we classified HIIT according to the exercise modality. Our results show that the cardiorespiratory response induced by R-HIIT meets the definition of HIIT (>85%HRmax). However, disparities in oxygen uptake and metabolic characteristics between the two HIIT modalities indicate that there might be differences in physiological adaptation. In addition, although some studies have suggested that R-HIIT has advantages over traditional HIIT in improving muscle fitness (McRae et al., 2012; Buckley et al., 2015; Sheykhlouvand et al., 2022), R-HIIT might not be as good as traditional resistance training (RT) in increasing muscle mass and strength because RT allows for heavier loads and longer interval rest periods. Second, although our pilot experiment revealed that participants could perform two types of HIIT with similar perceived effort (RPE), the exercise intensity performed could not precisely match R-HIIT and C-HIIT due to the differences in the exercise forms. Another limitation is that we did not measure 1-repetition maximum to set the squat load but directly used 20% of the individual’s body weight, which may lead to various individual physiological responses during exercise because of differences in strength levels. Similarly, the $\dot{V}$ O_2_max and HRmax obtained from the maximal incremental test using cycling on cycle ergometer are lower than the values measured using running on the treadmill because of differences in the muscle mass utilized in each method (Miyamura and Honda, 1972). Therefore, the percentage of maximum oxygen consumption and maximum heart rate obtained in both HIIT protocols might be slightly higher than the values obtained by running on the treadmill. It can be presumed that the cardiorespiratory response induced by resistance-type HIIT would be lower than that of running HIIT under the same perceived effort. Furthermore, the health benefits of using a single lower extremity R-HIIT might be limited compared to whole-body R-HIIT. Further research is needed to create a more comprehensive R-HIIT program to better improve cardiovascular and muscle fitness. Lastly, this study included a homogenous sample group (normal body mass, young, and recreationally trained males). Thus, the findings cannot be generalized to a broader population. The impact of this R-HIIT protocol or modified version on other people (e.g., obese or overweight populations) warrants further investigation.

## 5 Conclusion

Our study demonstrates that the R-HIIT protocol can elicit similar cardiovascular, hormonal, and perceptual responses as C-HIIT but with a higher contribution to the anaerobic glycolysis energy system. In contrast, C-HIIT is superior to R-HIIT for increasing oxygen consumption during exercise. Therefore, the two types of HIIT may lead to different metabolic and neuromuscular adaptations. R-HIIT might be advantageous in simultaneously improving cardiovascular and muscle fitness.

## Data Availability

The original contributions presented in the study are included in the article/Supplementary Material, further inquiries can be directed to the corresponding author.

## Abbreviations

HR, Heart rate; HRmax, Maximal heart rate; HRmean, Mean heart rate; $\dot{V}$ O_2_, Oxygen consumption; $\dot{V}$ O_2_max, Maximal oxygen consumption; $\dot{V}$ O_2_mean, Mean oxygen consumption; C-HIIT, Cycling high-intensity interval training; R-HIIT, Resistance-type high-intensity interval training; HIIT, High-intensity interval training; MICT, Moderate-intensity continuous training; EE, Energy expenditure; RPE, Rating of perceived exertion; RPEpeak, Peak rate of perceived exertion; RPEmean, Mean rate of perceived exertion; Blac, Blood lactate concentration; HRV, Heart rate variability; PPO, Peak power output; lnRMSSD, Log-transformed root mean square of successive R-R intervals; lnHF, Log-transformed high frequency power; η^2^, Eta squared
